# Bridging the gap between GRACE and GRACE Follow-On by combining high–low satellite-to-satellite tracking data and satellite laser ranging

**DOI:** 10.1007/s00190-024-01888-5

**Published:** 2024-09-13

**Authors:** Matthias Weigelt, Adrian Jäggi, Ulrich Meyer, Daniel Arnold, Torsten Mayer-Gürr, Felix Öhlinger, Krzysztof Sośnica, Sahar Ebadi, Steffen Schön, Holger Steffen

**Affiliations:** 1 Institute for Satellite Geodesy and Inertial Sensing, German Aerospace Center (DLR), Callinstraße 30b, 30167 Hanover, Germany; 2 Institut für Erdmessung, Leibniz University of Hannover, Schneiderberg 50, 30167 Hanover, Germany; 3 Astronomical Institute, University of Bern, Sidlerstraße 5, 3012 Bern, Switzerland; 4 Institute of Geodesy, Graz University of Technology, Steyrergasse 30/III, 8010 Graz, Austria; 5 Institute of Geodesy and Geoinformatics, Wroclaw University of Environmental and Life Sciences, Grundwaldzka 53, 50-375 Wrocław, Poland; 6 Lantmäteriet, Lantmäterigatan 2C, 80182 Gävle, Sweden

**Keywords:** Time-variable gravity, HL-SST, SLR, GRACE, GRACE Follow-On, Mass estimation, Hydrology, Glacial isostatic adjustment

## Abstract

The satellite missions GRACE and GRACE Follow-On have undoubtedly been the most important sources to observe mass transport on global scales. Within the Combination Service for Time-Variable Gravity Fields (COST-G), gravity field solutions from various processing centers are being combined to improve the signal-to-noise ratio and further increase the spatial resolution. The time series of monthly gravity field solutions suffer from a data gap of about one year between the two missions GRACE and GRACE Follow-On among several smaller data gaps. We present an intermediate technique bridging the gap between the two missions allowing (1) for a continued and uninterrupted time series of mass observations and (2) to compare, cross-validate and link the two time series. We focus on the combination of high-low satellite-to-satellite tracking (HL-SST) of low-Earth orbiting satellites by GPS in combination with satellite laser ranging (SLR), where SLR contributes to the very low degrees and HL-SST is able to provide the higher spatial resolution at an lower overall precision compared to GRACE-like solutions. We present a complete series covering the period from 2003 to 2022 filling the gaps of GRACE and between the missions. The achieved spatial resolution is approximately 700 km at a monthly temporal resolutions throughout the time period of interest. For the purpose of demonstrating possible applications, we estimate the low degree glacial isostatic adjustment signal in Fennoscandia and North America. In both cases, the location, the signal strength and extend of the signal coincide well with GRACE/GRACE-FO solutions achieving 99.5% and 86.5% correlation, respectively.

## Introduction

The launch of the Gravity Recovery And Climate Experiment (GRACE) mission (Tapley et al. 2004) in 2002 induced a new era in time-variable gravity field (TVG) recovery. GRACE observations allowed for observation of global mass changes down to wavelengths of $\approx 300~\hbox {km}$ for 15 years. It officially ended with the decommissioning of GRACE-2 in October 2017. The successor mission GRACE Follow-On (GRACE-FO) was launched on May 22^nd^, 2018. First products became available starting from June 2018 resulting in a data gap of approximately one year between the two missions. Bridging gaps by other techniques ensures a continuous time series which is especially important for the monitoring of nonlinear and non-periodic events such as floods, drought or unusually strong ice melts among others. We focus here on high–low satellite-to-satellite tracking (HL-SST) observations derived from Global Positioning System (GPS) to low-Earth orbiting (LEO) satellites in combination with satellite laser ranging (SLR) observations.

The primary sources for HL-SST solutions are kinematic positions and their covariance information which are limited by the precision of Global Navigation Satellite Systems (GNSS) observations which is approximately three orders of magnitude worse compared to the K-band observations of the GRACE mission. In this paper, we use the term *GPS* instead of *GNSS* since all observed kinematic positions are derived from GPS observations, only. TVG recovery using HL-SST has been attempted since the start of the CHAllenging Minisatellite Payload (CHAMP)-mission (Reigber et al. 2002), but with very limited success (Sneeuw et al. 2003; Moore et al. 2005). The first realistic TVG solutions were achieved by Prange (2010) using stacked solutions allowing for the derivation of the mean annual signal. Weigelt et al. (2013) used the same kinematic orbit data and adopted a Kalman filtering scheme yielding multi-year trends and annual signals for very long-wavelength TVG (spatial scales of approximately 2000 km). Based on this solution, Baur (2013) determined mass changes for Greenland that only differed by 10% from GRACE estimates. Visser et al. (2014) and Jäggi et al. (2015) retrieved TVG using Gravity Field and steady-state Ocean Circulation Explorer (GOCE) observations but stated that the recovery using solely this satellite remains challenging. Recently, a refined data weighting scheme allowed Arnold et al. (2023) to co-estimate trends and annual variations till degree and order 10. Jäggi et al. (2016) and Bezděk et al. (2016) recovered long-wavelength features of the TVG based on kinematic orbits of the Swarm satellites which are similar in quality as GRACE-based HL-SST solutions. Another Swarm-only TVG time series has been derived by Lück et al. (2018). In most cases, the underlying kinematic orbits are derived by linearly combining frequencies of the GPS observations. Zehentner and Mayer-Gürr (2016) followed a different approach avoiding those linear combinations and concluded that deriving TVG solutions from a number of satellites allows for the assessment of temporal variations in the gravity field. Zhou et al. (2020) discussed the benefit of HL-SST constellations for gravity field recovery. Teixeira da Encarnação et al. (2016) showed the benefit of combining different kinematic orbit products for the Swarm satellites. Teixeira da Encarnação et al. (2019a) derived the TVG solutions from Swarm by combining orbit observations from different processing centers and using various approaches. They are regularly provided as Swarm-only monthly solutions of the International Combination Service for Time-Variable Gravity Fields (COST-G) (Jäggi et al. 2022) which are one possibility to bridge the gap between GRACE and GRACE-FO.

SLR on the other hand has been a substantial contributor to the recovery of the longest wavelengths, typically degree 2–5, of the gravity field since the launch of Starlette in 1975 and LAGEOS-1 in 1976 (Pearlman et al. 2019a). Ultra-short and precise laser pulses are used to achieve a tracking precision of a few millimeters for so-called normal points (Degnan 1993). The technique benefits from the generally simple construction of passive satellites being dense and spherical in shape and thus having a small area-to-mass ratio. This minimizes orbit perturbations due to non-gravitational forces, e.g., atmospheric drag and solar radiation pressure. The number of SLR observations is limited by the need for a relative station-satellite visibility, weather conditions (clouds) and the inhomogeneous distribution of SLR stations. Currently, there are 38 SLR stations, 31 on the northern hemisphere and 7 on the southern hemisphere. The distribution causes a lack of SLR observations satellites over regions crucial to geophysical studies, e.g., Antarctica, Greenland, India and Central Africa. SLR tracking observations of geodetic satellites have been used for the recovery of the changes in the Earth’s oblateness (Cheng et al. 1997), changes in the Earth’s figure axis (Cheng et al. 2011), or temporal variations in the zonal spherical harmonics of the gravity field, which are associated with secular changes and long-term oscillations of the satellite orbital elements (Bianco et al. 1998; Cheng and Tapley 1999). Till today, the quality of SLR-derived $\bar{C}_{20}$ variations is better than GRACE-based values because of a strong aliasing of the $S_2$ tide into the GRACE solutions (Chen et al. 2009). Cheng and Ries (2017) also suggest that the unexpected signal observed in GRACE estimates of $\bar{C}_{20}$ may be attributed to a semi-diurnal and latitudinal behavior in the cross-track component of the GRACE accelerometer data which represent the non-gravitational forces affecting $\bar{C}_{20}$ estimates, as well as strong correlations between $\bar{C}_{20}$ and $\bar{C}_{40}$. SLR solutions show a remarkably reduced impact of the background modeling deficiencies and in particular of those related to the $S_2$ tide, since observations to satellites at different altitudes and inclination angles are assimilated. The SLR-derived TVG parameters contain significant seasonal variations up to about degree and order 6. Sośnica et al. (2015) and Gałdyn et al. (2024) showed that with SLR seasonal variations and secular changes in the Earth’s gravity field due to, e.g., ice mass loss in Antarctica or the melting of the Patagonian glaciers are observable on a similar level of precision as derived from GRACE but with a lower spatial resolution with the maximum expansion up to degree 10.

A first combination of HL-SST and SLR has been described in Meyer et al. (2019) where Swarm kinematic orbit data have been combined with SLR data for the period from January 2015 to June 2016 in order to test the possibility of bridging the gap between GRACE and GRACE Follow-On. Compared to K-Band-based GRACE solutions, the combined gravity fields match significantly better in the overlapping time period. SLR-only solutions slightly overestimate mass loss in Greenland but by the combination the RMS of the differences is reduced by almost 100 Gt.

In this paper, the work of Meyer et al. (2019) is vastly expanded considering the time span from 2003 to 2022 and data of 20 satellite missions. The CHAMP-mission was the first dedicated HL-SST for gravity field recovery. Each satellite in the pairs of GRACE and GRACE Follow-On are here considered as independent HL-SST-type satellites making no use of the ranging observations. Likewise, no gradiometer data are used in case of the GOCE mission. Additionally, we use data of 16 non-dedicated satellite missions, namely the Meteorological Operational Satellites MetOp-A and MetOp-B, TerraSAR-X, TanDEM-X, the three satellites of the Swarm mission, Jason 1 to 3, and Sentinel 1A, 1B, 2A, 2B, 3A and 3B. We use kinematic orbit data from four different processing centers: 1) the Astronomical Institute of the University of Bern, 2) the Institute of Geodesy at the Technical University Graz, 3) the Institut für Erdmessung at Leibniz University of Hannover (Swarm-only) and the Technical University Delft (Swarm-only and distributed by the European Space Agency) totaling 46 kinematic orbit products for the aforementioned satellites. The HL-SST data are combined with SLR observations to nine satellites, namely LAGEOS-1 and -2, AJISAI, LARES, Beacon-C, Starlette, Blits, Stella and Larets, on the normal equation level. The combined TVG solution is further improved by applying temporal filtering of the spherical harmonic coefficients using the Kalman filter approach introduced by Kurtenbach et al. (2012) for daily GRACE solutions. We present mass change estimates for various regions and estimates of the glacial isostatic adjustment signal in Fennoscandia and North America. With the available time series, we do not only bridge the gap between GRACE and GRACE-FO but also fill smaller data gaps present in the timeline of both mission solutions.

We first introduce the processing steps in Sect. 2 with emphasis on the combination in Sect. 2.3. Subsequently, high-frequency noise in the time series of the spherical harmonic coefficients requires temporal filtering of the spherical harmonic coefficients to improve the estimates of TVG signals, which we outline in Sect. 2.3.2. We compare our results with GRACE and GRACE-FO and mass trends for Greenland, the Amazon and the Danube basin in Sect. 3.1. Mass rate estimates for Fennoscandia and North America that exhibit the GIA signal are derived in Sect. 3.2.

## Gravity field recovery processing strategy

The generation of TVG solutions from combined HL-SST and SLR data requires the following steps:

1. generating normal equations for HL-SST considering pre-elimination of technique specific parameters,
2. generating normal equations for SLR with likewise pre-elimination,
3. determining of relative weights,
4. solving the combined equation system,
5. iteration (optional), and
6. temporal filtering (optional).

The normal equations for both techniques, described for HL-SST in Sect. 2.1 and for SLR in Sect. 2.2, are generated separately. Since we are only interested in gravity field parameters, data-specific parameters, e.g., calibration parameters for accelerometer data in case of HL-SST or range biases in case of SLR, are pre-eliminated. Various relative weighting schemes for the combination are discussed in Sect. 2.3.1. Depending on the chosen scheme, iteration may or may not be required. Finally, we introduce a Kalman environment in Sect. 2.3.2 for temporal filtering allowing for a significant reduction of the noise level.

### HL-SST gravity field determination

The HL-SST gravity field recovery employed here is a two-step procedure: the position for each satellite is first estimated in a purely geometrical way. These so-called kinematic positions are connected in a second step to a gravity field quantity. Several possibilities and approaches exist, e.g., the energy balance approach, the acceleration approach or the short-arc method. All yield very similar results with the exception of the energy balance approach (Baur et al. 2014). We consider here data spanning the period from January 2003 till December 2022 which allows us to bridge the gap between GRACE and GRACE-FO but also allows us to fill the various data gaps during the missions. Data availability changes constantly depending on the mission lifetime of the various satellites and the provision of data by the aforementioned processing centers.

#### Kinematic orbit determination

Kinematic positions are calculated and provided by various processing centers. Here, we use kinematic orbit products provided by the Astronomical Institute of the University of Bern, the Institute of Geodesy at the Technical University Graz, the Technical University Delft, and the Institut für Erdmessung at the Leibniz University of Hannover.

Astronomical Institute, University of Bern Kinematic orbit positions are computed in a batch least-squares adjustment using an ionosphere-free GPS carrier phase observation approach, e.g., Jäggi et al. (2016). Pseudo-range measurements are used for the initial orbit determination and receiver clock synchronization with GPS time. In the first step, the phase data are screened for outliers and cycle slips and new carrier phase ambiguity parameters are set up where necessary. For GPS-based orbits of high quality, the application of Phase Center Variation (PCV) maps is essential (Jäggi et al. 2009). Usually, these maps are generated by an iterative stacking of carrier phase residuals of a reduced-dynamic precise orbit determination over an extended time span. Kinematic orbit determination is then performed by estimation of epoch-wise three-dimensional positions and receiver clock corrections, as well as carrier phase ambiguity parameters. While the entire variance–covariance information for kinematic positions and clock corrections can be provided, usually only epoch-wise variance–covariance values are stored and used. The entire processing is performed using the development version of the Bernese GNSS Software (Dach et al. 2015). Kinematic orbit products^1^ are provided for GOCE, GRACE, GRACE-FO, Sentinel 1A, 1B, 2A, 2B, 3A and 3B, and the three Swarm satellites.

Institute of Geodesy, Technical University Graz The Institute of Geodesy follows a different approach avoiding linear combination or differences. As a drawback, errors that are normally eliminated or mitigated by the former need to be either known *a priori* or parameterized within the least-squares adjustment. The advantage is that the noise level remains unchanged as the observations are used as is. New observables, e.g., L5 signals, can be used without further modifications as no frequency-specific combinations are formed. The approach is referred to as raw measurement approach and is described in detail by Zehentner and Mayer-Gürr (2016) who also demonstrate the applicability of the derived kinematic orbits for HL-SST gravity field recovery. Kinematic orbit products^2^ are provided for CHAMP, GOCE, GRACE, GRACE-FO, Jason 1–3, MetOp-A and MetOp-B, Sentinel 1A, 1B, 3A and 3B.

Technical University Delft The official orbit level 2 Precise Science Orbit (PSO) product for the Swarm mission is computed by the Faculty of Aerospace Engineering at Delft University of Technology in the framework of the Swarm Satellite Constellation Application and Research Facility (SCARF) (van den IJssel et al. 2015) and distributed by the European Space Agency.^3^ Details on the processing are given in Montenbruck et al. (2018). Nominally, two types of orbit solutions, a reduced-dynamic and a kinematic orbit, are provided, whereas only the kinematic orbit product is of interest for gravity field recovery. The impact of possibly poor observations is reduced by requiring a minimum number of six available GPS observations per epoch. For both orbits, the POD strategy is based on an undifferenced approach and uses a standard Bayesian weighted least-squares estimator.

*Institut für Erdmessung, Leibniz University of Hannover* The approach is based on the precise point positioning (PPP) technique (Zumberge et al. 1997). The approach requires a detailed analysis of the tracking performance and the repairing of cycle slips to reduce the number of ambiguities. Melbourne-Wübbena and ionosphere-free linear combinations are used. The kinematic orbit is then determined in a standard least-squares adjustment. Orbit products for Swarm only are available on request (Le Ren and Schön 2018; Le Ren 2021).

#### Acceleration approach

For the derivation of the gravity field, we employ the acceleration approach, e.g., Reubelt et al. (2006), to determine HL-SST gravity field solutions from the kinematic positions on a monthly basis. Teixeira da Encarnação et al. (2019a) showed that also the combination of multiple approaches is beneficial but we focus here on a single approach to reduce the computational effort. We show that the results are on the same level as the combined solutions, i.e., the benefit of combining various approaches is compensated by the larger number of observations. Improvements might therefore be possible by using the larger number of observations with various approaches.

In the acceleration approach, the first step is to form pseudo-observations by double-differentiation of the kinematic positions $\textbf{r}$ yielding accelerations $\ddot{\textbf{r}}$ which can be directly connected to the gradient of the gravity field using Newton’s equation of motion. The methodology is known as the (point-wise) acceleration approach for HL-SST data processing (Reubelt et al. 2006). The basic equation in the inertial frame reads:

1 $$\begin{aligned} \ddot{\textbf{r}} = \nabla V + \textbf{f}_\textrm{3rd body} + \textbf{f}_\textrm{tides} + \textbf{f}_\mathrm {rel.} + \textbf{f}_\mathrm {non-grav.} + \textbf{f}_\mathrm {grav.}, \end{aligned}$$

where $\nabla V$ is the gradient of the Earth gravitational potential *V*. All other forces acting on the satellite need to be reduced from the observed accelerations $\ddot{\textbf{r}}$, e.g., third-body related forces $\textbf{f}_\textrm{3rd body}$, tidal forces $\textbf{f}_\textrm{tides}$, relativistic corrections $\textbf{f}_\mathrm {rel.}$, and non-gravitational forces $\textbf{f}_\mathrm {non-grav.}$ as the sum of atmospheric drag, solar radiation pressure, Earth Albedo, and other non-gravitational forces, which can be either modeled or observed by an onboard accelerometer. Furthermore, time-variable gravitational changes $\textbf{f}_\mathrm {grav.}$ with frequencies higher than one month should be reduced to avoid aliasing, e.g., using atmospheric and ocean de-aliasing products. Table 1 gives an overview of the applied reduction models. The six-hourly spherical harmonic coefficients of the atmospheric and ocean de-aliasing (AOD) product are linearly interpolated to the epoch of observation. We make use of the accelerometer data where suitable, namely for the missions CHAMP, GRACE, GRACE-FO and GOCE and co-estimated accelerometer biases and scale factors. For the second satellite of GRACE-FO, we use the accelerometer transplant data provided by the Institute of Geodesy, Technical University Graz. The accelerometer data of Swarm A and Swarm B have been compromised by the influence of the surrounding temperature. For simplicity, we do not use the accelerometer data of any Swarm satellite but compensate the primary influence of non-gravitational forces by co-estimating daily empirical constant accelerations in the satellite reference frame, i.e., in the radial, along-track and out-of-plane direction. Likewise, we treat all other high-low satellite missions without accelerometers onboard. All satellite specific parameters are pre-eliminated before combination using a rigorous block-inversion method.

**Table 1:** Background models for the HL-SST processing

Source	Model
Third-body forces	Point masses for Sun, Moon and the planets coordinates from DE440 (Park et al. 2021)
Solid Earth tide	IERS2010 (Petit and Luzum 2010, §6.1)
Pole tide	IERS2010 (Petit and Luzum 2010, §6.2)
Ocean tide	FES2014b (Lyard et al. 2021)
Ocean pole tide	IERS2010 (Petit and Luzum 2010, §6.3)
Atmospheric tide	AOD1B Release 06 (Dobslaw et al. 2017)
De-aliasing product	AOD1B Release 06 (Dobslaw et al. 2017)
Relativistic corr	IERS2010 (Petit and Luzum 2010, §10.2)

The gravitational potential *V* is developed into a spherical harmonic (SH) expansion till degree and order 60 (Hofmann-Wellenhof and Moritz 2006). The unknown SH coefficients $\bar{C}_{lm}$ and $\bar{S}_{lm}$ are estimated in a standard least-squares adjustment without regularization. The stochastic model is arguably the most crucial point for a successful combination of various satellite missions as it governs the coefficient-dependent relative weighting. Since the kinematic orbit products are typically provided epoch-wise neglecting correlations between epochs, the resulting error estimates are often too optimistic. It is therefore mandatory to iteratively improve the stochastic model during the gravity field recovery based on the estimated residuals. We employ the method described in Ellmer (2018) to co-estimate power spectral densities and arc-wise weight factors for each satellite and axis by variance-component estimation (VCE). The pseudo-observations are rotated to the local north-oriented frame (LNOF). Each axis as well as data from different processing centers is considered independent. This is a simplifying assumption which may result in biased or optimistic error estimates as it is often the case when neglecting correlations. Hardware limitations in the handling of large data sets does not allow for considering these kind of correlations at the current stage but may be subject to further investigations in future.

**Fig. 1:**
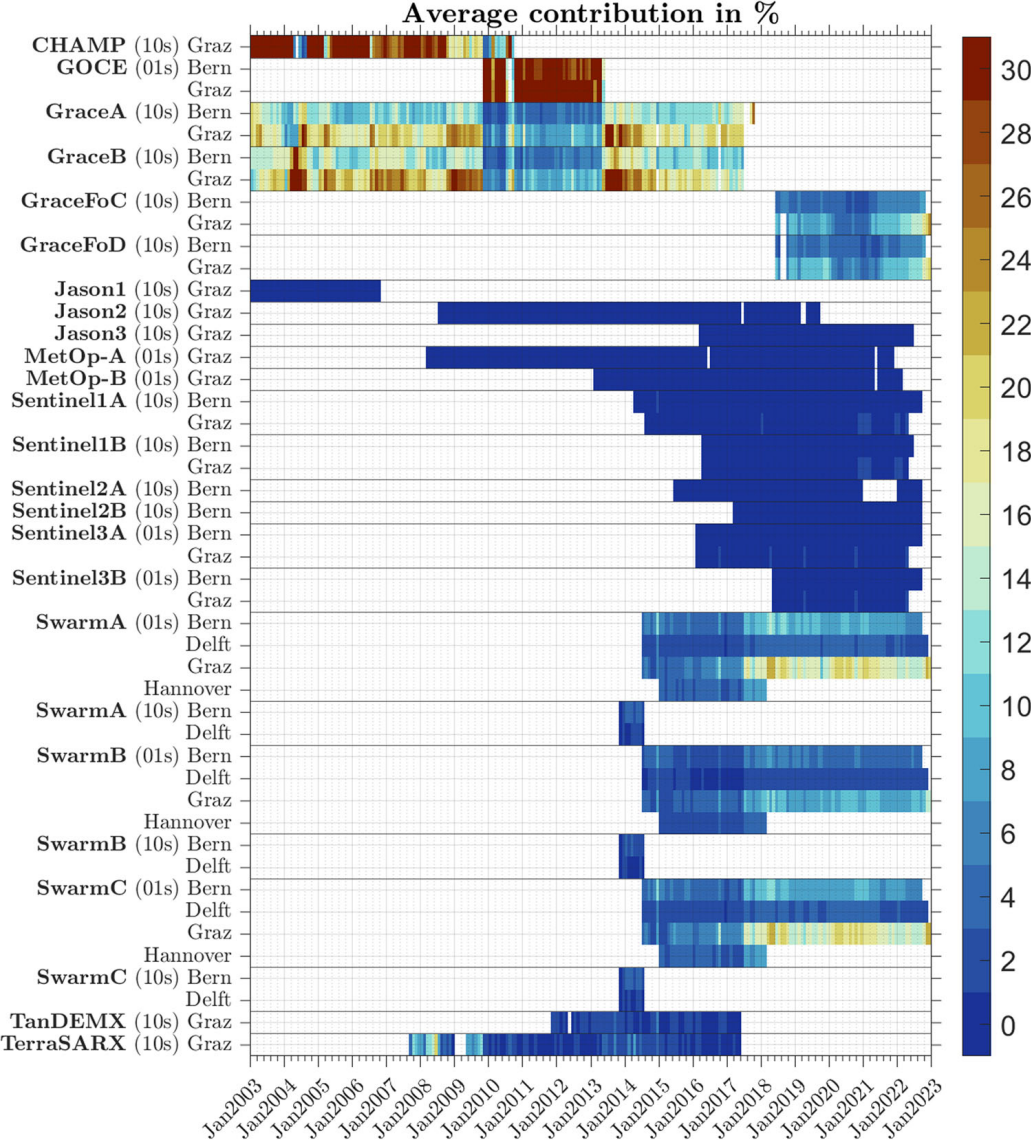
Data availability and relative contribution to each monthly solution

Figure 1 shows color-coded and in percentage the average relative contribution of each data set to the solution for each month. The relative contribution of a particular data set *i* can be calculated by

2 $$\begin{aligned} \textbf{R}_i = \textbf{N}^{-1}\textbf{N}_i \end{aligned}$$

where $\textbf{N}_i$ is the normal matrix corresponding to the data set *i*, $\textbf{N}$ is the accumulated normal matrix over all data sets, i.e., $\textbf{N} = \sum _i \textbf{N}_i$, and $\textbf{R}_i$ the relative contribution of this data set (Sneeuw 2000). Obviously, $\sum _i \textbf{R}_i = \textbf{I}$ holds. We extract then the diagonal elements of $\textbf{R}_i$ which have the same ordering as the unknown coefficients and average over all spherical harmonic parameters to determine their average contribution.

Generally, the main contributors are the CHAMP and GRACE mission in the early years, GOCE for the period 2009 till 2013, again GRACE till its decommissioning in the mid of 2017, the Swarm mission, and GRACE-FO starting from the mid of 2018. During the data gap between GRACE and GRACE-FO, the backbone of the solutions is the Swarm data. The primary reason for the observed variations is the orbit height acknowledging that the aforementioned averaging process over all estimated spherical harmonic coefficients is impacting the results as well. A lower orbit height yields higher contributions to the solution which is especially obvious during the availability of the GOCE data. Being at an orbit height of $\approx 250~\hbox {km}$, it dominates the solution from 2009 till 2013. Other missions such as Jason, MetOp, Sentinel, TerraSAR-X and TanDEM-X are typically in orbit heights of $600~\hbox {km}$ to $1500~\hbox {km}$ and contribute only to the very low degrees. The benefit is masked here by the mentioned averaging process.

Besides the orbit height, the quality of the GPS receivers, the availability and quality of attitude information and the processing strategy have a significant impact on the contribution of data sets. The latter can be seen when comparing the contributions of the Swarm data of the various processing centers to the monthly solutions during the data gap between GRACE and GRACE-FO from mid-2017 till mid-2018. The primary source of the solution is the data provided by the Technical University Graz with approximately 15%-20%, followed by the Astronomical Institute of the University of Bern with 10% to 15%, the Leibniz University of Hannover, with 5%-10% and the TU Delft with less than 5% for each satellite. Also the impact of the higher orbit of Swarm B relative to Swarm A and Swarm C is visible.

### SLR data processing

SLR range observations can be employed for gravity field recovery using a dynamic approach and deriving corresponding normal equations. In SLR solutions, we use observations to two high-orbiting LAGEOS satellites (at the altitudes of 5800 km and 5600 km, for LAGEOS-1 and LAGEOS-2, respectively) and up to seven low-orbiting satellites: AJISAI (altitude: 1500 km), LARES (1440 km), Beacon-C (940–1300 km), Starlette (800–1100 km), Blits (820 km), Stella (810 km), and Larets (690 km). The data are available from the International Laser Ranging Service^4^ (ILRS) (Pearlman et al. 2019a, b; Noll et al. 2018). Additional data and background models needed for a reliable analysis of SLR observations are provided by the EUROLAS Data Center.^5^ The availability of SLR observations varies in time for different satellites, e.g., Blits was launched in September 2009, but in January 2013 Blits disintegrated into pieces after a collision with space debris (Kelso et al. 2013). LAGEOS-1 had the smallest area-to-mass ratio among all artificial satellites until February 2012. Now, LARES is the densest object in the Earth’s orbit with the smallest area-to-mass ratio. Beacon-C is the only non-spherical satellite used in the SLR solutions (Pearlman et al. 2019a). Despite its larger sensitivity to non-gravitational orbit perturbations compared to spherical satellites, we decided to include this satellite into our solution due to its low inclination angle ($41^{\circ }$) and non-circular orbit with a large eccentricity which help to decorrelate some of the gravity field parameters. The largest contribution to the multi-satellite SLR solutions emerges from LAGEOS-1, LAGEOS-2, LARES, Starlette, Stella, and AJISAI due to the very high precision of their orbits. The contribution of other satellites is downweighted as they typically introduce some modeling problems or are characterized by insufficient number of observations.

For high-orbiting LAGEOS-1/2, we generate 10-day orbital arcs, while for low-orbiting SLR satellites 1-day arcs are generated. The LAGEOS satellites allow recovering zonal gravity field parameters, which are associated with the long-term orbit perturbations, whereas the tesseral and sectorial harmonics benefit most from low-orbiting SLR satellites which are related to relatively small-scale amplitude and high-frequency oscillations in the satellite orbits. For low-orbiting satellites the a priori atmosphere density model NRL-MSISE-00 (Picone et al. 2002) is applied along with a constrained estimation of once-per-revolution empirical parameters in the along-track direction. In the out-of-plane direction, the empirical parameters are strictly constrained to zero due to strong correlations with $\bar{C}_{20}$. For all SLR satellites except LAGEOS-1/2, 14-daily pseudo-stochastic pulses in the along-track direction are co-estimated to compensate for the impact of atmospheric drag.

Sośnica et al. (2014) found that the estimation of the pseudo-stochastic pulses for low-orbiting satellites improves the SLR solutions by about 30%. Generating short 1-day arcs and estimating pseudo-stochastic pulses for low-orbiting SLR satellites prevent the accumulation of the orbital errors in the estimated gravity field parameters and allow recovering non-zonal harmonics.

We simultaneously estimate the gravity field parameters along with SLR station coordinates, satellite orbits, range biases for low-orbiting satellites, and Earth rotation parameters (pole coordinates and length-of-day). This is dictated by the link of SLR solutions to the terrestrial reference frame whereas the fine structures of the gravity field changes are derived from small-scale orbit perturbations. It is necessary to consider epoch-wise nonlinear station motions. Deficiencies in station coordinate modeling may lead to accumulation of various systematic effects in the gravity field parameters, similar to the effects in the estimated station coordinates when using just a static a priori gravity field model (Zelensky et al. 2014). Sośnica et al. (2014) found that the simultaneous estimation of $\bar{C}_{20}$ and length-of-day is particularly beneficial for the length-of-day parameter. It reduces the offsets and formal errors by a factor of 12–13 compared to the SLR solution without co-estimating gravity field parameters.

Ries and Cheng (2014) suggest that for the recovery of finer-scale gravity field features with full amplitudes, the SLR solutions should be estimated with a spherical harmonic expansion to at least degree 7. In this paper, the SLR gravity field solutions are estimated up to degree and order 10 for the subsequent combination with the HL-SST solutions. The large number of estimated parameters (gravity field coefficients, orbit parameters, station coordinates, range biases, Earth rotation parameters), compared to a limited number of available SLR observations, leads to the over-optimistic assessment of the formal errors of SLR-derived gravity field parameters. Moreover, some of the gravity field parameters are strongly correlated with each other, e.g., $\bar{C}_{21}$ and $\bar{C}_{61}$ (Ries and Cheng 2014) or $\bar{C}_{30}$ and $\bar{C}_{50}$ (Sośnica et al. 2014). The correlations can be mitigated to some extent by using the satellites of different inclination angles and different altitudes or by splitting normal equations (Gałdyn et al. 2024).

The SLR gravity field solutions are generated using the development version of Bernese GNSS Software (Dach et al. 2015). For a consistency with HL-SST solutions, we apply the same modeling conventions for pole tides, solid Earth tides, ocean pole tides, and relativistic effects based on the IERS Conventions 2010. We apply EOT11a ocean tide model and the atmospheric and ocean dealiasing (AOD) corrections. All parameters except for gravity field parameters are pre-eliminated with previously applied no-net-rotation minimum constraint on the subset of core SLR stations following the recommendations of the International Laser Ranging Service (Pearlman et al. 2002). A detailed description of the SLR gravity field solutions can be found in Sośnica et al. (2015).

### Combination and post-processing

For the combination of the observations from both observations techniques, we choose a three-steps procedure (c.f. Sect. 2.3.1) with a fourth optional step on temporal filtering (c.f. Sect. 2.3.2):

1. Observations for each satellite are preprocessed and combined on the observation level forming normal equations for each observation technique separately.
2. Technique specific parameters like, e.g., accelerometer biases for HL-SST or station coordinates for SLR among others are pre-eliminated.
3. Combining the normal equations of the two techniques using a relative weighting scheme.

The procedure is a consequence of processing the data of the two observation techniques with two different software tools. SLR data are processed with Bernese GNSS Software (Dach et al. 2015). HL-SST data are processed with the so-called GRAvity Software Processing (GRASP) developed at the Institut für Erdmessung, Leibniz University of Hannover. This is an artificial limitation as the relative weighting within each technique is co-estimated but the relative weighting between HL-SST and SLR remains unknown. Processing both data sets in a common framework is desirable.

#### Combination on the normal equation level

The task of combining two or more observation techniques on normal equation level reduces to a weighted summation of the normal matrices $\textbf{N}_k$ and the vector $\textbf{y}_k$, where *k* refers here to HL-SST and SLR, respectively.

3 $$\begin{aligned} \textbf{N}= &  \sum \limits _{k} \frac{1}{\sigma ^2_k} \textbf{N}_k \nonumber \\ \textbf{y}= &  \sum \limits _{k} \frac{1}{\sigma ^2_k} \textbf{y}_k, \end{aligned}$$

where $\sigma ^2_k$ are the variances of the data sets. Typically, relative weights $\omega _k = {\sigma ^2_k}/{\sigma ^2_1}$ are introduced and the problem reduces to the determination of a single weight factor $\omega$:

4 $$\begin{aligned} \textbf{N}= &  \textbf{N}_\mathrm {HL-SST} + \omega \, \textbf{N}_\textrm{SLR} \nonumber \\ \textbf{y}= &  \textbf{y}_\mathrm {HL-SST} + \omega \, \textbf{y}_\textrm{SLR}. \end{aligned}$$

From Eq. (4), it becomes obvious that the combination relies on a single weighting factor for all unknown spherical harmonic coefficients. The technique’s specific signal and noise behavior must therefore be realistically described within the normal matrix. We test three different methods for the determination of $\omega$:

1. unit weighting,
2. co-estimation by variance-component estimation (VCE)  (Koch and Kusche 2002),
3. empirical weight determination minimizing the ocean root mean square (RMS) under consideration of a $700~\hbox {km}$ Gaussian filtering.

**Fig. 2:**
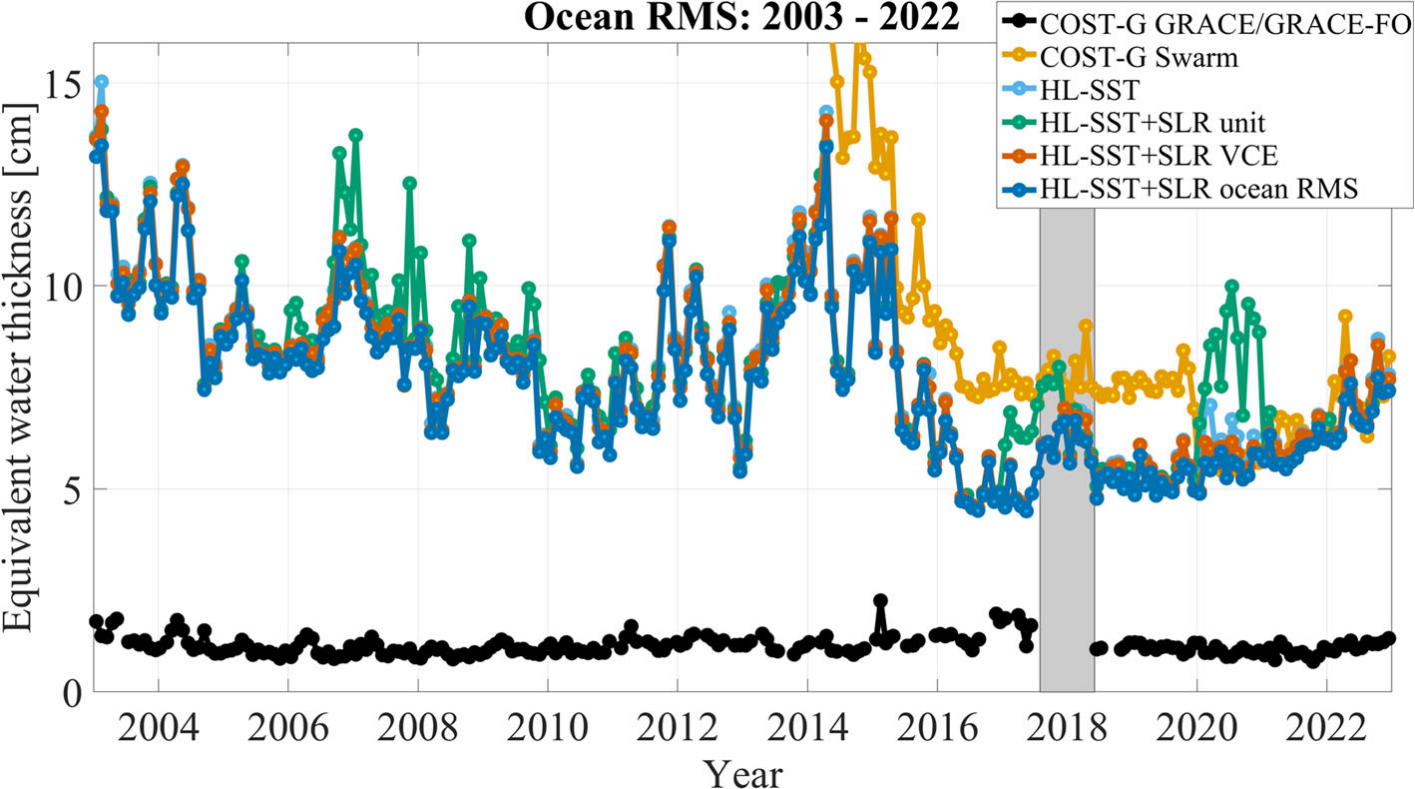
Ocean RMS for HL-SST-only and combined solutions in comparison to GRACE/GRACE-FO and Swarm solutions from COST-G. Gaussian filtering of $700~\hbox {km}$ has been applied

The ocean root mean square (RMS) is calculated as the root mean square over ocean areas after reducing the mean, trend, annual and semi-annual signal—the so-called climatology—from each grid cell of a $1^\circ \times 1^\circ$. A buffer zone of $200~\hbox {km}$ is used to reduce leakage from coastal land. Figure 2 shows the results of the combination as time series of ocean RMS values. The combined COST-G GRACE RL01 and GRACE-FO RL02 time series (Meyer et al. 2020; Jäggi et al. 2022; Meyer et al. 2023), hereafter called COST-G GRACE/GRACE-FO (black line), and the COST-G time series for Swarm (orange line) (Teixeira da Encarnação et al. 2019b) are added for comparison. For these, $\bar{C}_{20}$ and $\bar{C}_{30}$ have been replaced using SLR-estimates based on the technical note 14 (Loomis et al. 2020). The calculation of the ocean RMS requires the reduction of the climatology which has been extracted from the COST-G time series and consistently been removed from all shown solutions. The HL-SST-only solution (light blue line) is mostly hidden behind the combined solutions based on a unit weighting (green line), on VCE (red line) or the ocean RMS (blue line). This does not imply that the contribution of SLR to the solution is negligible, but the contribution is mostly limited to degree 2 as shown below.

The solution using unit weighting performs significantly worse than the HL-SST-only solution and the other combinations. The reason is found in the different stochastic modeling for both observation techniques. For HL-SST, we mentioned in Sect. 2.1.2 that we employed variance component estimation to iteratively estimate temporal correlation and thus improve the stochastic model (Ellmer 2018). Due to the excellent properties of the method, we can assume that the HL-SST data are providing realistic error information. For SLR observations, the situation is different. The limited number of observations requires to base the stochastic information on models. As a consequence, SLR tends to deliver over-optimistic *a posteriori* standard deviations due to strong correlations between the coefficients. A combination therefore unrealistically favors the SLR solution and the resulting solutions is degraded. A better approach is to use VCE which results in significantly more consistent solutions compared to the HL-SST-only solutions but occasionally still performs worse. Again the reason can be found in the same limited stochastic modeling in SLR. Further, the estimation is governed by the noise in the higher coefficients due to their poorer signal-to-noise ratio.

**Fig. 3:**
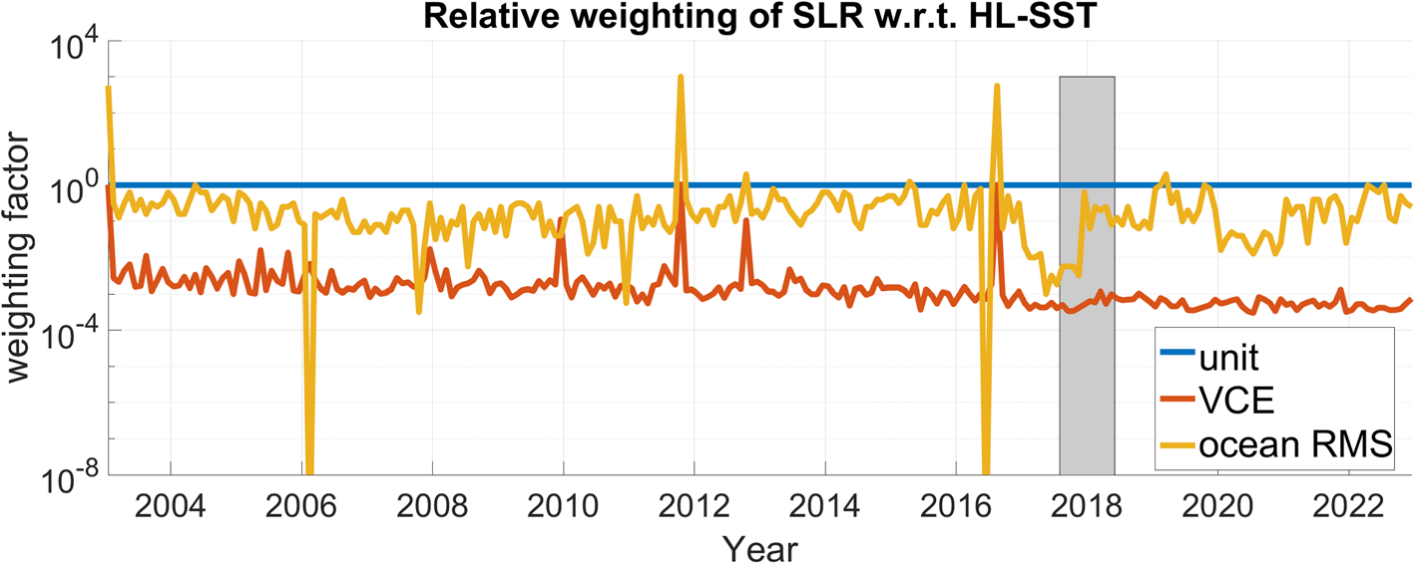
Weights of the SLR solution relative to the HL-SST solution for each month and for each combination approach

As a workaround, we developed an empirical approach which uses the ocean RMS as optimization criterion. The impact of high-frequency noise is reduced by smoothing the solution with a Gaussian filter with a radius of $1000~\hbox {km}$. In a brute-force approach, solutions are then calculated with weighting factors $\omega = 10^i$ where *i* is ranging from -10 to 10 in steps of 0.1. The solution with the lowest overall ocean RMS is selected. The result is depicted as light blue line in Fig. 2 and weighting factors are shown in Fig. 3.

Overall, Fig. 2 reveals correlations of the solutions with the solar cycle. Starting near the maximum of the solar cycle 23 in 2003, the solutions have ocean RMS values of $\approx 11~\hbox {cm}$ in terms of equivalent water height. It slowly decreases to $\approx 6~\hbox {cm}$ till the solar minimum in 2009 and early 2010. Afterward, the RMS is increasing in accordance with solar cycle 24 and reaches maximum values of $\approx 13~\hbox {cm}$ during 2014. Subsequently, values drop below $5~\hbox {cm}$ during the solar minimum in 2019 before slowly increasing since 2020 due to the ongoing solar cycle 25. Solutions are slowly improving over the period of 2003 till 2017 due to the decaying orbit of the GRACE satellites.

The period of the data gap between GRACE and GRACE Follow-On from October 2017 till June 2018 is marked as grayish area. Two interesting effects are observable. First, a significant increase in ocean RMS values of about $\approx 20\%-30\%$ is observable with the loss of the two GRACE satellites. This is due to higher orbit height of the Swarm satellites but more importantly due to the reduced number of observations. The opposite is happening with the advent of the two GRACE Follow-On satellites. The solutions do not reach the same low level as in 2016 and 2017 due to the higher orbits of the two GRACE Follow-On satellites compared to the low orbits of GRACE at the end of the mission. We conclude that orbit height and data availability have both equally important impacts on the quality of a solution. Secondly, HL-SST-only and combined solutions perform significantly better than the COST-G Swarm solution till 2020. The reason is a change in the processing of the Swarm data at Technical University Graz which significantly improved the quality of the solution (about a factor of two). Although these improved kinematic orbits are available for the entire mission period, they have only been adopted in the COST-G Swarm solutions from 2020 onward. The kinematic orbit product of Swarm of TU Graz is consequently dominating the solution which can also be seen in the relative contributions in Fig. 1. With the refined orbit product in use, the COST-G Swarm and the solutions presented here are aligned. It is acknowledged that the COST-G Swarm solution is an operational product and the adoption of changes does not propagate to the past at the current stage. However, it is reasonable, that (1) COST-G engages in a reprocessing of the entire time series for Swarm and (2) other processing centers close the gap to the kinematic orbit products to Graz to increase the benefit of combining solutions.

The weights of each of the three combination approaches are shown in Fig. 3 relative to the monthly HL-SST solution. Typically, *i* varies between $-3$ and $-2$ for the VCE method and between -2 and 0 for the empirical ocean RMS-based method with few exceptions for both. The unit weighting obviously gives higher weight compared to the other techniques. VCE solutions on the other hand are down-weighting SLR too strongly and the better approach is found in using the ocean RMS as weighting criterion. The empirical approach based on the ocean RMS shows consistent behavior compared to the VCE solutions on a higher level but is not flawless as visible in February 2006 and in May 2016. Here, the approach eliminates the SLR-contribution and the solutions for these two months are identical to the HL-SST-only solution. Marginal differences in the ocean RMS for all weighting factors compromised the selection of the numerical minimum resulting in the low weighting factor. The VCE solutions for these two month do not show a similar behavior and improvements for the empirical method might be achieved by replacing or introducing additional criteria, e.g., maximizing the signal on land areas. The primary effort should, however, be on improving the stochastic modeling for SLR data.

**Fig. 4:**
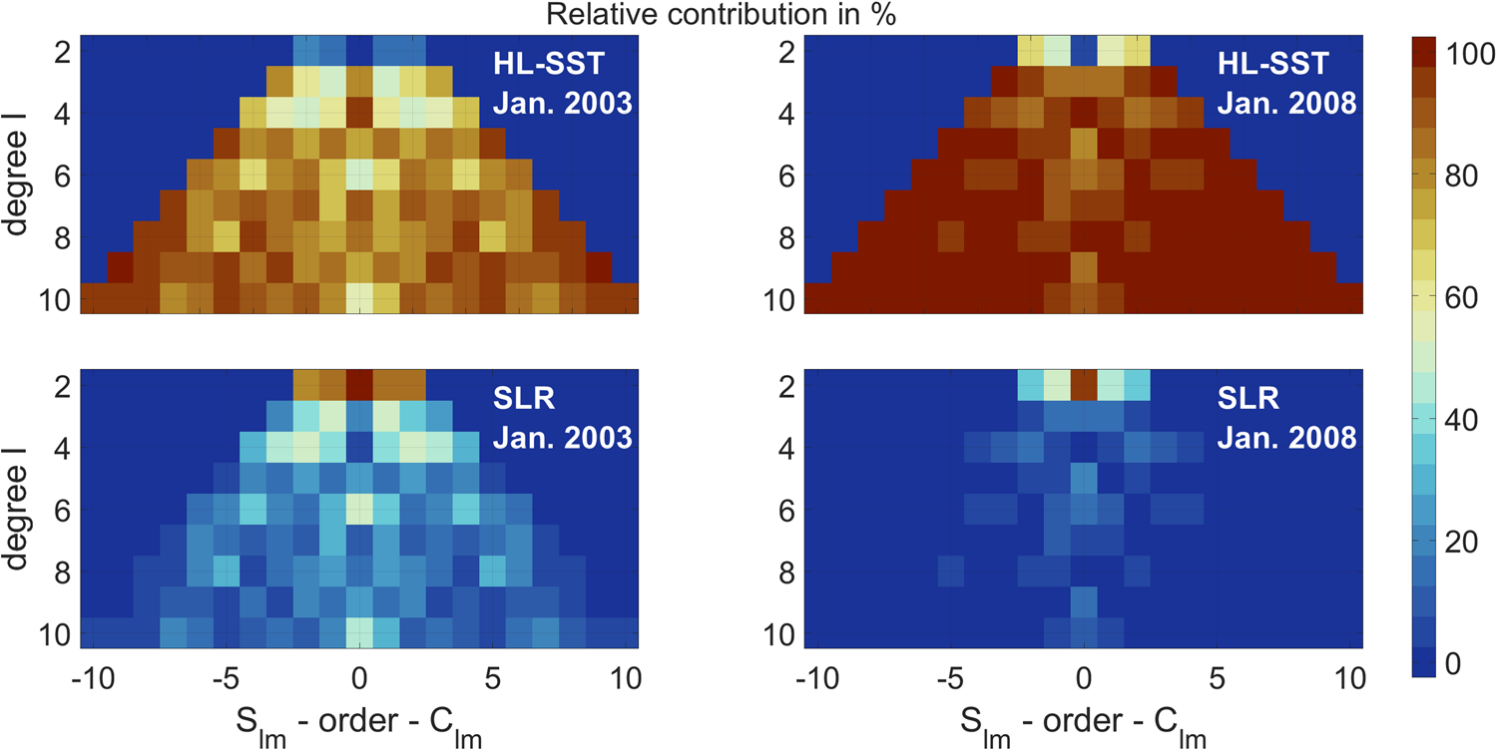
Exemplary contribution to the combined monthly solution for January 2003 and 2008

Figure 4 exemplarily shows the contribution of HL-SST and SLR to a monthly solution for January 2003 and 2008 derived using equation (2). Obviously, the contribution of each observation technique to the combined solution varies from month to month and depends on the derived weight. SLR primarily contributes to degree 2 coefficients, especially to the coefficient $\bar{C}_{20}$ which is almost fully determined by SLR. The contribution to the coefficient $\bar{C}_{30}$ is marginal. For GRACE and GRACE-FO solutions, the replacement of this coefficient with SLR is recommended for periods of impaired accelerometer data requiring the transplant of accelerometer data (Loomis et al. 2020; Cheng and Ries 2023). The improved transplant data render this recommendation superfluous. With increasing degree, the contribution of SLR is decaying quickly.

Based on the findings of this section, the empirical approach using the ocean RMS as optimization criterion is chosen as final result for the rest of this paper. The time series is available as QuantumFrontiers $\rightarrow$ HLSST_SLR_COMB2023 solution at the International Center for Global Earth Models (ICGEM) (Ince et al. 2019).

#### Temporal filtering

The final step of the processing chain is an optional step and comprises temporal filtering of the time series of the spherical harmonic coefficients. Weigelt et al. (2013) showed that the time series of each coefficient is compromised by high and low frequency noise which can be significantly reduced by filtering in the time domain. A Kalman filter-based approach was developed that efficiently deals with the limited number of samples minimizing the impact of undesirable filter properties (e.g., warm-up effects). This idea is refined here by following the approach of Kurtenbach et al. (2009, 2012). The strategy consists of two steps: (1) determination of a residual quantity by reducing the trend and the mean annual signal estimated from the available data in a least-squares adjustment and (2) Kalman filtering of the residual time series. The reduction of the trend and the mean annual and semi-annual signal is a prerequisite in the concept as the (residual) quantity needs to be describable by a stochastic process.

Generally, in a Kalman filter some predicted value is updated by observations which may come from various sources. Following the idea of Kurtenbach et al. (2012), the prediction step is given as:

5 $$\begin{aligned} \textbf{x}_t = \textbf{C}_\Delta \textbf{C}_0^{-1} \textbf{x}_{t-1} + \textbf{q}, \end{aligned}$$

where $\textbf{C}_0$ denotes the auto-covariance matrix and describes the spatial variability. $\textbf{C}_\Delta$ denotes the cross-covariance matrix and describes the variability between epochs. The process noise, $\textbf{q}$, is given as $\textbf{q} \sim N\left( 0,\textbf{Q}\right)$ where $\textbf{Q} = \textbf{C}_0-\textbf{C}_\Delta \textbf{C}_0^{-1}\textbf{C}_\Delta ^T$. It is implicitly defined by the auto- and cross-covariances and thus no ensemble approach as in Weigelt et al. (2013) is necessary. The covariance matrices of the temporal and spatial variations of the Earth gravity field are unknown and need to be approximated by empirical ones. All subsequent steps of the Kalman filter including the application of a Rauch–Tung–Striebel (RTS) smoother are identical to the procedure outlined in Kurtenbach et al. (2012).

For the derivation of the empirical covariance matrices, we follow the procedure of Kurtenbach et al. (2012) and use the ESA Earth System Model (Dobslaw et al. 2015) after reducing bias, trend, and annual and semi-annual signals from the time series of coefficients. Arguably, the usage of models in the processing include a risk to introduce unwanted a priori information, but we emphasize that the procedure does not introduce the models in a deterministic way, e.g., as pseudo-observation or via regularization. Instead, the average stochastic behavior of the model is used and in no earlier applications to GRACE data a bias to the applied model has been observed.

**Fig. 5:**
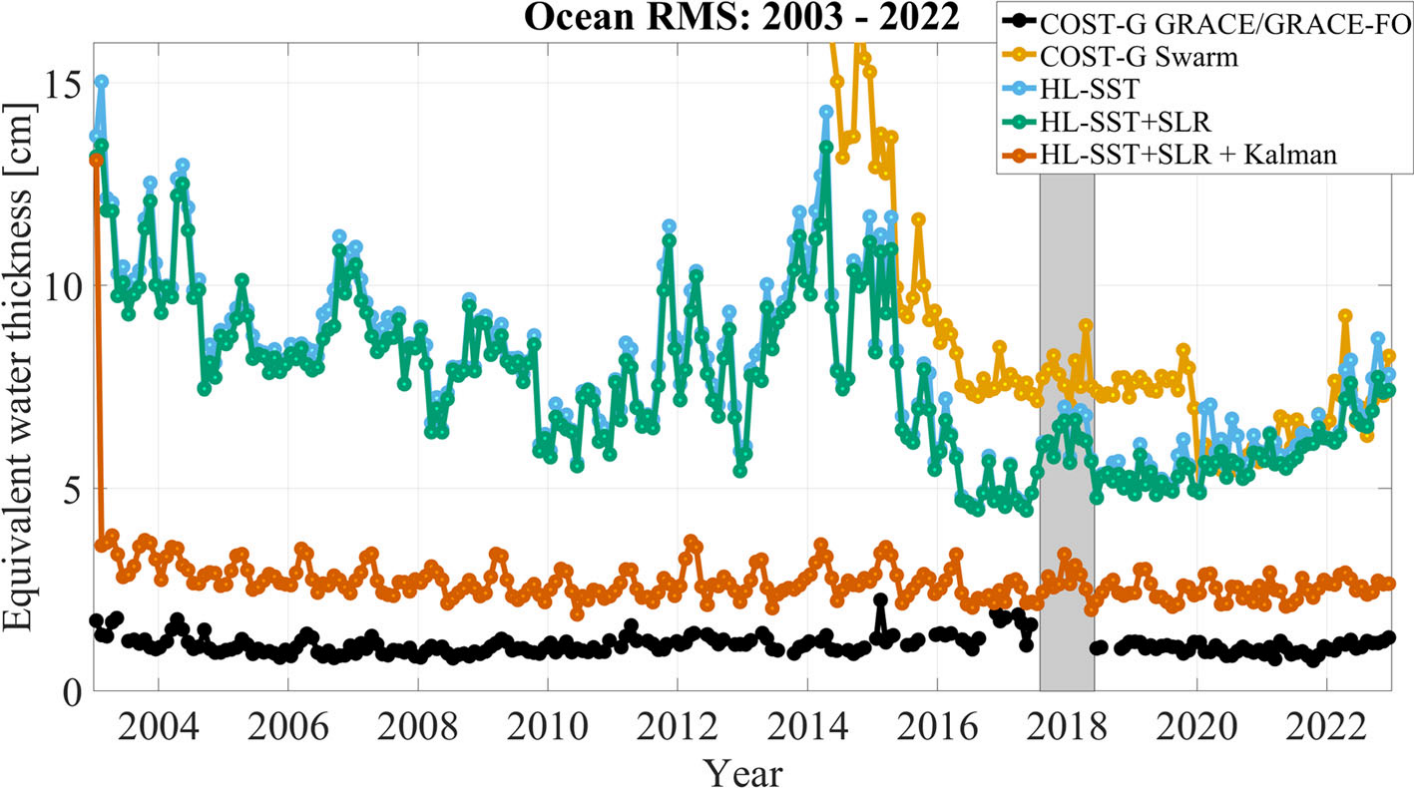
Ocean RMS for the combined HL-SST+SLR and Kalman filtered solutions in comparison to GRACE/GRACE-FO and Swarm solutions from COST-G. Gaussian filtering of $700~\hbox {km}$ has been applied

In Fig. 5, the Kalman-filtered solution (red curve) is shown in comparison to the COST-G GRACE/GRACE-FO (black line), the COST-G Swarm (orange line) and the HL-SST-only solution (light blue line), and the unfiltered HL-SST+SLR solution (green line). The peak in January 2003 is related to the warm-up effect of the Kalman filter. The ocean RMS level of the filtered solution is generally reduced by a factor 2–4. The correlation with the solar cycle is vastly reduced but some seasonality remains visible, especially in years of high solar activity. The ocean RMS remains on the level of 2–4 cm in terms of equivalent water height, which is about a factor 2–3 worse than the GRACE/GRACE-FO solution. Overall, the temporal filtering yields a significant improvement which not only reduces the noise level but also reveals time-variable signals beyond the climatology as demonstrated in the subsequent Sect. 3.1.

## Results and validation

The ocean RMS in Figs. 2 and 5 is a good approximation of the noise level of solutions but more importantly we are interested in the signal content of the solutions. Since HL-SST and SLR solutions have inherently less sensitivity than the ranging observations of GRACE and GRACE-FO, it is to be expected that the monthly solutions have a reduced spatial resolution. It can also be expected that the COST-G Swarm solution performs weaker compared to the HL-SST+SLR solutions due to a shorter time span and less satellite data. In Sect. 3.1, we quantify the observed time-variable gravity field solutions in comparison to GRACE and GRACE-FO time series and estimate the GIA signal in Fennoscandia and North America, c.f. Sect. 3.2.

### Time-variable gravity signals

#### Climatology

**Fig. 6:**
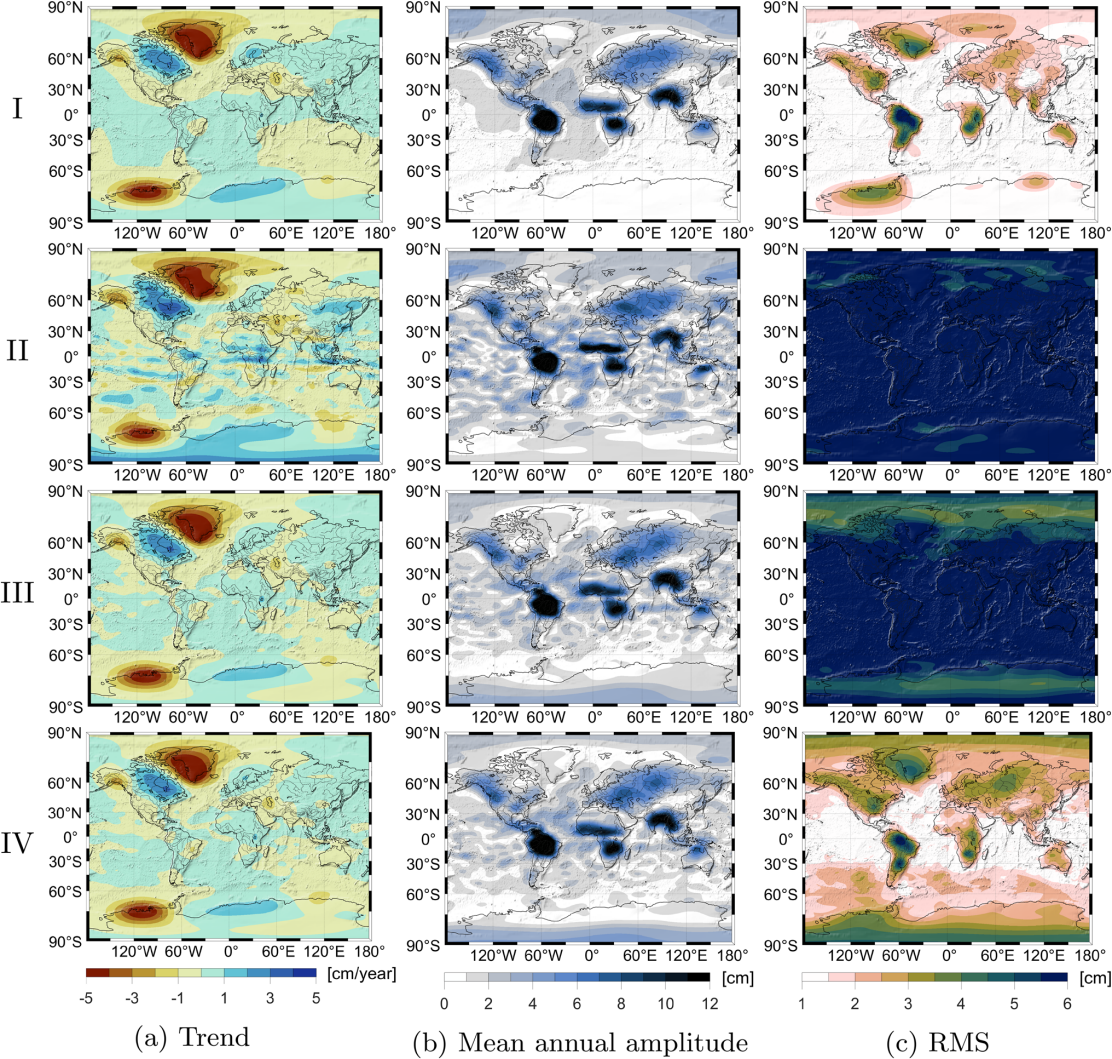
Trend (left column), mean annual amplitude (middle column) and root mean square of the residual signal after reducing trend, mean annual and semi-annual signals (right column) for the COST-G GRACE/GRACE-FO solution (I), the COST-G Swarm solution (II), the HL-SST+SLR solution (III) and the Kalman-filtered HL-SST+SLR solution (IV). All quantities are in terms of equivalent water height in $\hbox {cm}$ and Gaussian filtering of $700~\hbox {km}$ has been applied

The first test in Fig. 6 considers parts of the climatology, i.e., the trend (left column), the mean annual amplitude (middle column) and the root mean square of the residual signal (right column). The top row shows the COST-G GRACE/GRACE-FO solution which is considered the reference subsequently. The following rows show HL-SST solutions: first the COST-G Swarm solution (second row), the HL-SST+SLR solutions (third row) and the Kalman-filtered HL-SST+SLR solution (bottom row). All solutions have been filtered with a Gaussian filter of $700~\hbox {km}$, and for the COST-G solutions, $\bar{C}_{20}$ and $\bar{C}_{30}$ have again been replaced using SLR-estimates based on the technical note 14 (Loomis et al. 2020).

The trend signal in the left column shows primarily characteristic signals related to hydrology, like the loss of water in the Caspian Sea, in the south-eastern Amazon basin and in western North America, the decaying ice sheets on Greenland and western Antarctica, the glacier melt in Alaska and the increase in Fennoscandia and northeastern North America due to GIA. COST-G Swarm in the second row shows horizontal artifacts following the magnetic equator which is a known defect, c.f. Teixeira da Encarnação et al. (2019a), a decrease in Siberia and more noise in ocean areas. Both the unfiltered and Kalman-filtered HL-SST+SLR solutions can resolve these issues and show a much more consistent picture compared to the GRACE/GRACE-FO solution. A higher noise level remains visible in ocean areas. In polar areas, the solutions do not show the negative signal and positive signal at the North and South Pole, respectively, which might be an indication of over-smoothing. The ice accumulation area in eastern Antarctica is slightly deformed and of less extent indicating a smaller spatial resolution. The hydrological signal in central Africa is also slightly underestimated.

The mean annual signal (middle column) shows very similar results. The noise level of all HL-SST-based solutions is higher on the oceans with better performance of the HL-SST+SLR solutions compared to the COST-G Swarm. The latter can be explained by the outdated orbits used till 2020 in the COST-G Swarm solution. As mentioned in Sect. 2.1.2, the reason is a change in the processing of the Swarm data at Technical University Graz which significantly improved the quality of the solution (about a factor of two). Although these improved kinematic orbits are available for the entire mission period, they have only been adopted in the COST-G Swarm solutions from 2020 onward due to the nature of being an operational combination.

The Amazon basin is well resolved in all solutions with a peak value of $\approx 11-12~\hbox {cm}$. Significant differences can be seen at the west coast of North America. The GRACE/GRACE-FO solutions show an elongated area spanning the coast at a level of $\approx 5-6~\hbox {cm}$. The HL-SST solutions show a distinct peak in eastern Canada and less extension toward Alaska. Similar over-estimations and deformed areas can be observed in Siberia and northern Australia. In mid-western Africa, the two HL-SST+SLR solutions show a bulge extending northward which is better resolved in the COST-G solutions.

The right column shows the spatial RMS after subtracting the climatology. All calculations are done in the spatial domain. For each pixel, the trend, the mean annual and mean semi-annual signal are estimated in a least-squares adjustment and reduced from the time series of the pixel. The figures show then the RMS of the residual time series. It is immediately obvious that the COST-G Swarm solution and the unfiltered HL-SST+SLR solution have a much higher noise level consistent with the description in Sect. 2.3.2 and only the Kalman-filtered solution is able to recover significant portions of the residual signal content, e.g., the dual peaks in the Amazon basin and southern Africa and the peaks in Greenland and eastern North America. In Siberia the signal is overestimated as well as in the northern and southern latitudes of the ocean areas and eastern Antarctica. Nevertheless, the benefit of the Kalman filtering becomes obvious, when comparing the HL-SST+SLR solution with and without it.

**Table 2:** Statistics of the difference of all HL-SST-based solutions w.r.t. the COST-G GRACE/GRACE-FO solution for the trend, mean annual amplitude and the RMS shown in Fig. 6

Trend $[\hbox {cm}/\hbox {year}]$	Maximum	Minimum	Mean	RMS
COST-G Swarm	2.9	$-$2.2	0.0	0.6
HL-SST+SLR	0.4	$-$0.7	0.0	0.2
HL-SST+SLR+Kalman	0.5	$-$0.8	0.0	0.2

Mean Annual Amplitude $[\hbox {cm}]$	Maximum	Minimum	Mean	RMS
COST-G Swarm	5.8	$-$4.4	0.8	1.4
HL-SST+SLR	5.2	$-$3.0	0.5	1.1
HL-SST+SLR+Kalman	4.1	$-$2.3	0.5	1.1

RMS $[\hbox {cm}]$	Maximum	Minimum	Mean	RMS
COST-G Swarm	14.0	2.0	6.7	7.0
HL-SST+SLR	10.1	1.4	5.3	5.6
HL-SST+SLR+Kalman	3.3	$-$1.7	0.7	1.0

Table 2 shows the statistics of the difference of the three HL-SST-based solutions, i.e., the COST-G Swarm, the HL-SST+SLR and the Kalman-filtered HL-SST+SLR solutions in rows 2 to 4 of Fig. 6 with respect to the COST-G GRACE/GRACE-FO solution in the first row. An improvement is achieved by the HL-SST+SLR solutions compared to the COST-G Swarm solution as it is currently provided. The Kalman filtering process is slightly affecting the trend signal resulting in increased values, which is an indication that the filtering process can be further optimized. The mean annual amplitude improves in both the mean and the RMS of the difference, by approximately 50%. This is the smallest observable improvement and the COST-G Swarm is despite its shorter time period already providing a respectable estimate of the mean annual amplitude. We again point out that the results for the COST-G Swarm solution will improve as soon as the improved kinematic orbits are adopted for the entire Swarm mission period. It is expected that both, the HL-SST+SLR and COST-G Swarm solution perform similarly, except that the HL-SST+SLR solution benefits from a longer time series. Last but not least, the difference in the RMS is a good description of the noise level of the solutions. The benefit of the filtering process is clearly demonstrated as the RMS level of unfiltered HL-SST+SLR solution is reducing from 5.6 to $1.0~\hbox {cm}$ the one of the Kalman-filtered solution.

#### Time series for selected basins

**Fig. 7:**
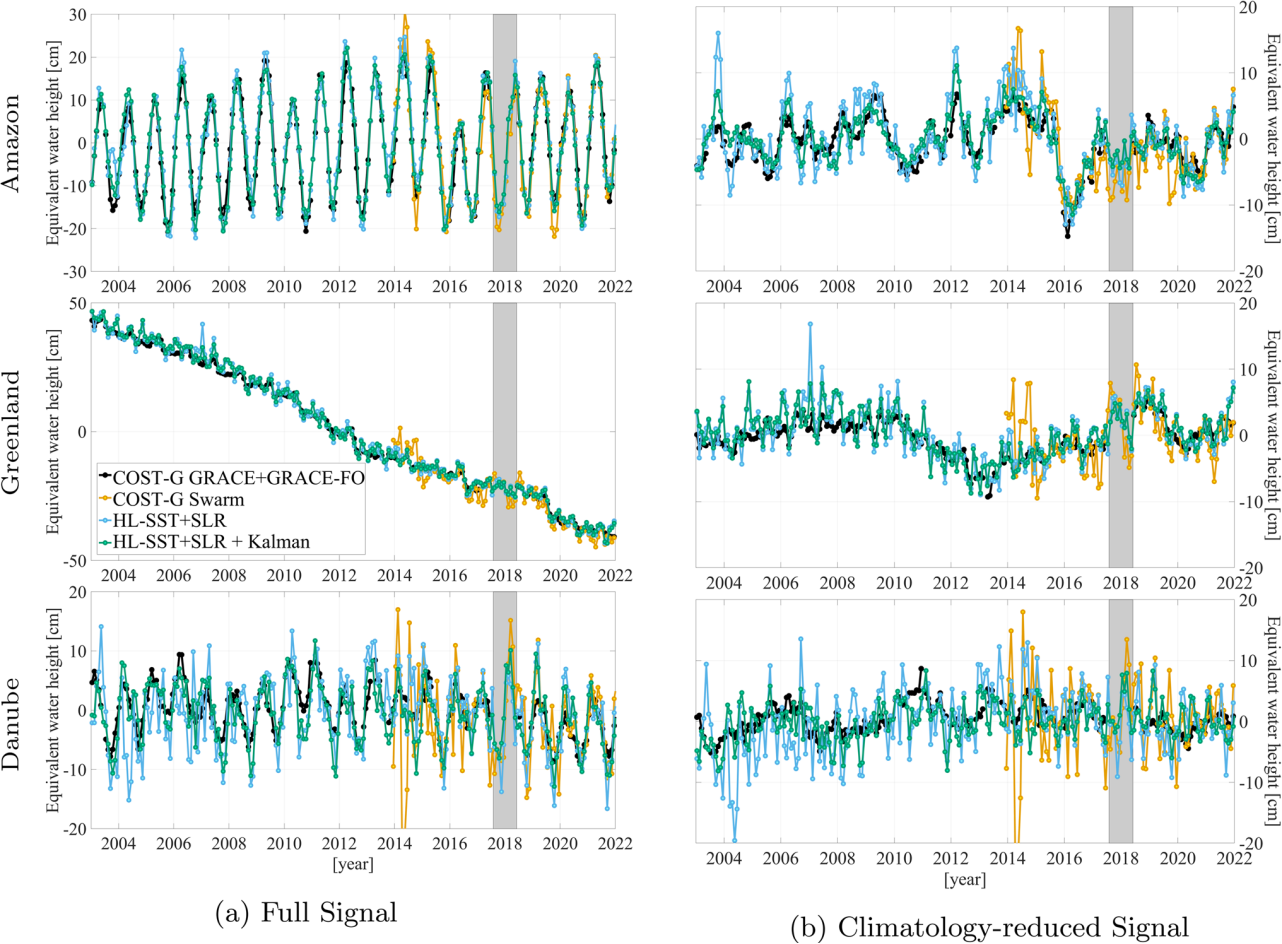
Time series of selected basins in equivalent water height: full signal (left column) and climatology-reduced signal (right column) for the Amazon basin (top row), Greenland (second row), and the Danube basin (bottom row). All quantities are in $\hbox {cm}$ and Gaussian filtering of $700~\hbox {km}$ has been applied. The gray areas mark the period of the data gap between GRACE and GRACE-FO

Section 3.1.1 discussed trends and mean signals of the time series. In this section, we investigate the temporal behavior for selected basins, namely the Amazon basin, containing the strongest hydrological signal, Greenland with the strongest ice melt signal, and the Danube basin in central Europe, which is challenging for HL-SST-based solutions with its reduced spatial resolution. Figure 7 shows the time series for the three basins in each row. The left column contains the full signal. In the right column, the climatology, which has been extracted from the COST-G GRACE/GRACE-FO time series, is consistently removed from all solutions. Please note the different scales of the y-axis in the left column.

In the Amazon basin, a good agreement can be expected and is observed between all solution due to the strong signal. Generally, all HL-SST-based solutions agree well with the COST-G GRACE/GRACE-FO time series. The solutions benefit from the large area of the Amazon basin as the determination of basin time series involves an averaging operator additionally smoothing noise. We also observed that for larger basins, smaller radii for the Gaussian filter are feasible (not shown here). The optimal filter radius is therefore a compromise between the noise of a solution, the basin size and the anticipated noise level of the basin time series. For this paper, we settled for a filter radius of $700~\hbox {km}$ as a compromise between noise suppression, spatial resolution and visual presentation. This filter radius is also used throughout the paper to allow for a consistent comparison and discussion, but users are advised to test the best filter radius for their specific applications.

The climatology-reduced signal for the Amazon basin in the right column shows subtle difference between the solutions. The HL-SST+SLR tends to overshoot the inter-annual variations and performs similarly as the COST-G Swarm solution. The latter shows significant oscillations in the period 2017 to 2020. With the adoption of the aforementioned improved kinematic orbits of the Technical University Graz, the oscillations settle and the solutions are in good agreement. The Kalman-filtered HL-SST+SLR solution again performs best due to its reduced noise level. In the early years, the solution also tends to overshoot the reference curve of the combined COST-G GRACE/GRACE-FO in blue. With the advent of GOCE data in late 2009, the agreement significantly improves and the curves follows closely the reference. Remarkably, all three HL-SST-based solutions are able to reveal the extreme drought in the Amazon basin in 2016 related to El Niño (Rodrigues 2023).

In Greenland, all solutions show the strong ice melting. Again the HL-SST+SLR and the COST-G Swarm solution show more oscillations and overshooting. The Kalman-filtered HL-SST+SLR solution agrees best with the reference solution. All three solutions show the significant melting flanks in the summer of 2012, 2016 and 2019 which proves the benefit of using observations from HL-SST and SLR to bridge the gap between GRACE and GRACE-FO. These nonlinear and non-periodic events are not observable otherwise. In the data gap period from June 2017 to June 2018, no such behavior is observable and the data indicate a normal melting period during the summer. The climatology-reduced signal on the right shows stronger oscillations for all HL-SST-based solutions than in the Amazon basin. The smaller area size (Greenland is about half the size of the Amazon basin) results in less smoothing in the determination of the basin time series. Also, better agreement is achieved if more data, e.g., during the GOCE period, are available.

**Table 3:** Statistics of the climatology-reduced signal shown in Fig. 7

Amazon $[\hbox {cm}]$	Maximum	Minimum	Mean	RMS
COST-G GRACE/GRACE-FO	6.9	$-$1.5	$-$0.5	3.7
COST-G Swarm	16.4	$-$12.1	$-$0.9	5.9
HL-SST+SLR	15.8	$-$13.4	$-$0.1	5.1
HL-SST+SLR+Kalman	10.6	$-$12.0	$-$0.1	3.7

Greenland $[\hbox {cm}]$	Maximum	Minimum	Mean	RMS
COST-G GRACE/GRACE-FO	5.1	$-$8.4	$-$0.3	2.8
COST-G Swarm	9.6	$-$10.0	$-$0.6	4.0
HL-SST+SLR	16.5	$-$9.7	$-$0.5	4.0
HL-SST+SLR+Kalman	8.4	$-$9.5	0.4	3.5

Danube $[\hbox {cm}]$	Maximum	Minimum	Mean	RMS
COST-G GRACE/GRACE-FO	8.5	$-$5.0	0.3	2.4
COST-G Swarm	17.8	$-$29.3	0.5	6.7
HL-SST+SLR	13.7	$-$19.0	$-$0.2	5.4
HL-SST+SLR+Kalman	8.3	$-$8.1	$-$0.1	3.1

**Fig. 8:**
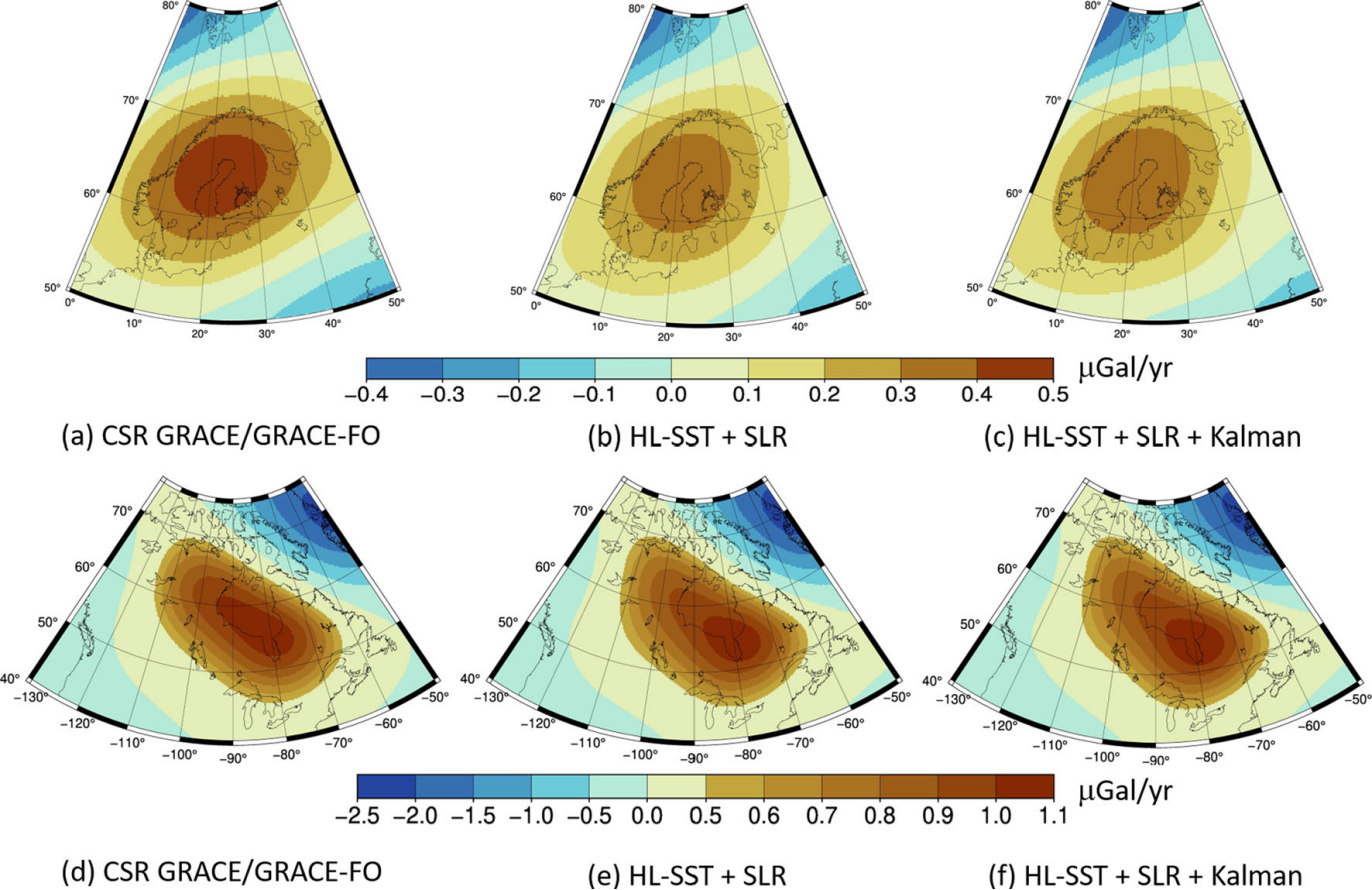
Trend estimates in Fennoscandia (top panel) and North America (bottom panel) from the HL-SST+SLR solution for the time span and the CSR GRACE/GRACE-FO Release 06 monthly solutions for the time span 01/2003–12/2002 using Gaussian filters of $700~\hbox {km}$. Units are in $\mu \hbox {Gal}/\hbox {year}$

The Danube basin is with $800000~\hbox {km}^{2}$ the smallest basin considered here. Anticipating to resolve degree 15 in terms of spherical harmonics, corresponding to $1300~\hbox {km}$ spatial resolution (half wavelength), of the time-variable gravity signal, the basin is beyond the expected spatial resolution of the monthly gravity field solutions. Nevertheless, the solutions still reveal some significant signal content. The Kalman-filtered solution again performs best showing the smallest differences in the climatology-reduced graph on the right. During the period of the data gap, the HL-SST-based solution shows a significant peak which may be related to a flooding event in spring 2018 in central Europe. However, a similar peak is observed about one year later in 2019 with approximately the same magnitude. The available GRACE-FO time series does not show any such peak, thus rendering the observation of the flood in the Danube basin in 2018 questionable. Beyond, similar observations regarding the relation of oscillations and the averaging area can be made.

Table 3 shows the statistics of the climatology-reduced signal depicted in the right column of Fig. 7. In the Amazon basin, the COST-G Swarm and the HL-SST+SLR solution show with $5.9~\hbox {cm}$ and $5.1~\hbox {cm}$, respectively, a higher variability in the RMS compared to the Kalman-filtered HL-SST+SLR with $3.7~\hbox {cm}$. The latter coincides with the GRACE/GRACE-FO solution confirming the higher noise level of the unfiltered HL-SST solutions. Both HL-SST+SLR solutions show a slight positive offset in the mean value. In Greenland all solutions perform equally well but again a noise reduction by the Kalman filtering can be observed in the extreme values as well as in the RMS. In the Danube basin, the smaller basin size challenges the spatial resolution capability of the COST-G and both, the unfiltered and Kalman-filtered, HL-SST+SLR solution. The RMS deviates significantly from the one of the GRACE/GRACE-FO solution, whereas the Kalman filtering is again reducing the noise-induced variability.

### Glacial isostatic adjustment

In this section, we attempt to estimate the GIA signal from the solutions. The GIA signal is a dominant long-wavelength signal in North America affecting all Canada east of the Rocky Mountains. In northern Europe, i.e., the Scandinavian Peninsula, Finland, Denmark, the Baltic States and north-west Russia, the signal is much smaller than in North America but still clearly visible in the trends (Steffen et al. 2010). We test how our solution performs in North America and northern Europe in comparison to the recent GRACE/GRACE-FO CSR Release 06 product. We select this solution due to its generally low noise level (e.g., Chen et al. 2021).

The postprocessing follows that of Steffen et al. (2010): gravity values $dg\left( \varphi , \lambda , t\right)$ are computed on a $1^{\circ }\times 1^{\circ }$ grid for each available month. Thereafter, a simultaneous fit of a constant, a linear trend, and up to four different periodicities is applied to the derived gravity data. The first periodicity is the annual trend. The second is a 2.5-yr period defined as an average of basin-related and filter-dependent frequency analysis (Schmidt et al. 2008). The last three periods are only applied to the CSR product and are due to aliasing for the S2, K2 and K1 tides (Ray et al. 2003). They result in 161-d, 3.7-yr and 7.4-yr periods, respectively. The solutions have then been filtered with a Gaussian filter of $700~\hbox {km}$ as in the previous section.

Figure 8 shows the calculated trends for the two areas and the two solutions. The signal in North America is located between $240^{\circ }$ and $300^{\circ }$ longitude and $45^{\circ }$ and $70^{\circ }$ latitude. The GIA signal can be captured well by the HL-SST+SLR solutions. There is agreement in the spatial extend but the location of the maximum is slightly shifted to the south-east. At the given resolution of $\approx 700~\hbox {km}$, all solutions cannot distinguish the two large domes of the former Laurentian ice complex (Tamisiea et al. 2007) but show an extension of the maximum area to the west of Hudson Bay, where the other maximum is expected. The maximum magnitude of our solutions is with approximately $1~\mu \hbox {Gal}/\hbox {year}$ about the same as that of CSR (Table 4) and a correlation analysis yields 99.5%, thus an excellent match.

In Fennoscandia, the area of discussion is found between $5^{\circ }$ and $45^{\circ }$ longitude and $55^{\circ }$ and $72^{\circ }$ latitude. The combined solutions show a smaller magnitude than the CSR solution, whereas the maximum is at about the same location, which is observed by GRACE/GRACE-FO to be in the Gulf of Bothnia near the Kvarken area, which is the shortest distance between Sweden and Finland. The shape in the HL-SST+SLR solutions is rather circular with slight distortion in the NW-SE direction, which does not agree with current knowledge of the GIA pattern in Fennoscandia: a typical elliptic pattern in SW-NE direction (Steffen et al. 2010). The correlation to GRACE/GRACE-FO is 86.5%, which is lower than the correlation result in North America.

**Table 4:** Location (in $^{\circ }$) of the maxima and trend estimates (in $\mu \hbox {Gal}/\hbox {year}$)

Solution	SE of Hudson Bay		Fennoscandia	
	lat/lon	$\dot{g}$	lat/lon	$\dot{g}$
CSR GRACE	56/278	1.08	64/22	0.48
HL-SST+SLR	54/281	1.08	64/21	0.39
HL-SST+SLR+Kalman	54/281	1.09	64/20	0.40

Both, the unfiltered and the Kalman-filtered HL-SST+SLR solutions perform equally as the trend estimation is a low-pass filtering process by itself. Kalman filtering can be disregarded for trend estimation although it does not significantly distort the signal. Thus, both the unfiltered and Kalman-filtered solutions have been published to offer users the best flexibility for their specific applications.

## Conclusion

In this paper, we presented an extended combined HL-SST+SLR time series for the determination of the time-variable gravity field with a monthly temporal resolution and an average spatial resolution of $700~\hbox {km}$. The time series is available as QuantumFrontiers $\rightarrow$ HLSST_SLR_COMB2023 solution at the International Center for Global Earth Models in an unfiltered and with optional temporal Kalman filtering version. Both allow for bridging the gap between GRACE and GRACE-FO. The covered time period from 2003 till end of 2022 also enables the closure of smaller gaps in the GRACE time series due to hibernation periods necessary to compensate the reduced battery functionality. The technique can be applied to similar periods for GRACE-FO and future satellite missions.

SLR contributes primarily to degree 2 coefficients, whereas HL-SST provides the higher spatial resolution. We compare to the COST-G Swarm solution as the best HL-SST based product so far. The presented solutions here outperform the COST-G Swarm solution in terms of stronger signal agreement and reduced noise level till 2020. Using the improved Swarm orbit product of the Technical University Graz from this time onward aligns the COST-G and the present HL-SST+SLR solution. The dominance of the kinematic orbit product of the TU Graz implies the opportunity to improve the orbit products of other processing centers further benefiting the combination. We also showed that the orbit height and data availability are equally important for the quality of a solution. Improvements in the COST-G Swarm are possible if more satellites beyond Swarm are included. Likewise, the presented HL-SST+SLR solutions should consider the combination of data processed with various approaches.

The combination of HL-SST and SLR solutions depends on proper stochastic modeling for both observation techniques. The best combination is here achieved by developing an empirical approach based on the ocean RMS as optimization criterion. We showed that this method is not flawless and recommend that the stochastic modeling in SLR solutions should be improved. Adopting the method of Ellmer (2018) is challenging due to the limited number of observations. The integrated processing in a single software package is a further desirable objective as currently hardware limitations require a separate processing resulting in neglected correlations between kinematic orbit products, and HL-SST and SLR observations.

Temporal filtering as implemented here in a Kalman environment yields a significant improvement. It reduces the noise level revealing time-variable signals beyond the climatology. As shown in Table 2, the Kalman filtering is slightly affecting trend signals and a further optimization is possible and desirable. Additional optimization might also be achieved by further harmonizing the background models. For the HL-SST data processing, the GRASP software used the FES2014b ocean tide model, whereas in the SLR data processing the EOT11a model has been used. Although we do not expect significant changes to the solution, consistent processing is always desirable and reduces possible error sources.

Overall, HL-SST+SLR solutions prove to contain valuable gravity field signals at the cost of a reduced spatial resolution. Although it is most desirable to have GRACE-like missions as the primary source to observe the time-variable gravity signal, HL-SST and SLR concepts and scenarios should be constantly considered in the planning of future satellite missions, e.g., as add-on to mission focusing on other purposes. Both techniques improve the redundancy of gravity field observations and serve as important and cost-effective source for comparison, validation and combination. The potential of HL-SST solutions to augment GRACE-like systems in terms of spatial coverage should also be investigated.

## Data Availability

The kinematic orbit products of the Astronomical Institute, University of Bern, can be found at ftp://ftp.aiub.unibe.ch/LEO_ORBITS, last accessed on June 3rd, 2023. The kinematic orbit products of the Institute of Geodesy, Technical University Graz, can be found at ftp://ftp.tugraz.at/outgoing/ITSG/satelliteOrbitProducts/operational, last accessed on May 25th, 2023. The kinematic orbit products of the Technical University Delft are distributed by the European Space Agency and can be accessed at ftp://swarm-diss.eo.esa.int, last accessed on May 28th, 2023. The kinematic orbit products of the Institut für Erdmessung, Leibniz University of Hannover, are available on request. Please contact Steffen Schön schoen@ife.uni-hannover.de. Data and background models for satellite laser ranging are provided by the ILRS https://ilrs.gsfc.nasa.gov/data_and_products/index.html and the EUROLAS Data Center at https://edc.dgfi.tum.de/en/ilrs-ac/. The data sets generated and analyzed during the current study are available in the ICGEM repository, http://icgem.gfz-potsdam.de/sp/03_other/QuantumFrontiers/HLSST_SLR_COMB2023s as well as from the corresponding author on reasonable request.

## Footnotes

1 ftp://ftp.aiub.unibe.ch/LEO_ORBITS.

2 ftp://ftp.tugraz.at/outgoing/ITSG/satelliteOrbitProducts/operational.

3 ftp://swarm-diss.eo.esa.int.

4 https://ilrs.gsfc.nasa.gov/data_and_products/index.html.

5 https://edc.dgfi.tum.de/en/ilrs-ac/.
